# Minerals and the Menstrual Cycle: Impacts on Ovulation and Endometrial Health

**DOI:** 10.3390/nu16071008

**Published:** 2024-03-29

**Authors:** Celine Kapper, Peter Oppelt, Clara Ganhör, Ayberk Alp Gyunesh, Barbara Arbeithuber, Patrick Stelzl, Marlene Rezk-Füreder

**Affiliations:** 1 Experimental Gynaecology, Obstetrics and Gynaecological Endocrinology, Johannes Kepler University Linz, Altenberger Strasse 69, 4040 Linz, Austria; celine.kapper@jku.at (C.K.); peter.oppelt@jku.at (P.O.); barbara.arbeithuber@jku.at (B.A.); 2 Department for Gynaecology, Obstetrics and Gynaecological Endocrinology, Kepler University Hospital, Johannes Kepler University Linz, 4020 Linz, Austria; 3 Division of Pathophysiology, Institute of Physiology and Pathophysiology, Medical Faculty, Johannes Kepler University Linz, 4020 Linz, Austria; 4 Clinical Research Institute for Cardiovascular and Metabolic Diseases, Medical Faculty, Johannes Kepler University Linz, 4020 Linz, Austria

**Keywords:** menstrual cycle, fertility, minerals, endometrial health, ovulation

## Abstract

The role of minerals in female fertility, particularly in relation to the menstrual cycle, presents a complex area of study that underscores the interplay between nutrition and reproductive health. This narrative review aims to elucidate the impacts of minerals on key aspects of the reproductive system: hormonal regulation, ovarian function and ovulation, endometrial health, and oxidative stress. Despite the attention given to specific micronutrients in relation to reproductive disorders, there is a noticeable absence of a comprehensive review focusing on the impact of minerals throughout the menstrual cycle on female fertility. This narrative review aims to address this gap by examining the influence of minerals on reproductive health. Each mineral’s contribution is explored in detail to provide a clearer picture of its importance in supporting female fertility. This comprehensive analysis not only enhances our knowledge of reproductive health but also offers clinicians valuable insights into potential therapeutic strategies and the recommended intake of minerals to promote female reproductive well-being, considering the menstrual cycle. This review stands as the first to offer such a detailed examination of minerals in the context of the menstrual cycle, aiming to elevate the understanding of their critical role in female fertility and reproductive health.

## 1. Introduction

Lifestyle factors such as weight, stress, smoking, and alcohol consumption can also significantly influence conception and the menstrual cycle [1,2,3]. This modern lifestyle, characterized by altered dietary habits, exposure to environmental pollutants, and increased stress, profoundly impacts an individual’s mineral status [4,5]. The exposure to heavy metals like lead and cadmium can antagonize mineral absorption and function, potentially worsening fertility challenges [6]. While the influence of minerals on male fertility has been intensively investigated [7,8,9,10], their role in women’s reproductive health and menstrual cycle has been marginally addressed, often only in the context of specific reproductive pathologies [11,12], emphasizing vitamins and multivitamin supplementation. Notably, these studies frequently lack in-depth investigation of the role of minerals and the underlying mechanisms, neglecting the phases of the menstrual cycle [13]. This review aims to address these gaps. We focus on individual minerals and their distinct roles in hormonal regulation, ovulation, oxidative stress, and endometrium health throughout the menstrual cycle. It provides a comprehensive overview of the role of minerals in female fertility and gives insights into potential therapeutic approaches for fertility challenges by offering evidence-based recommendations for mineral intake in reproductive-age women.

## 2. Hormonal Regulation and Fertility

### 2.1. Overview of Hormones and Fertility

**Figure 1:**
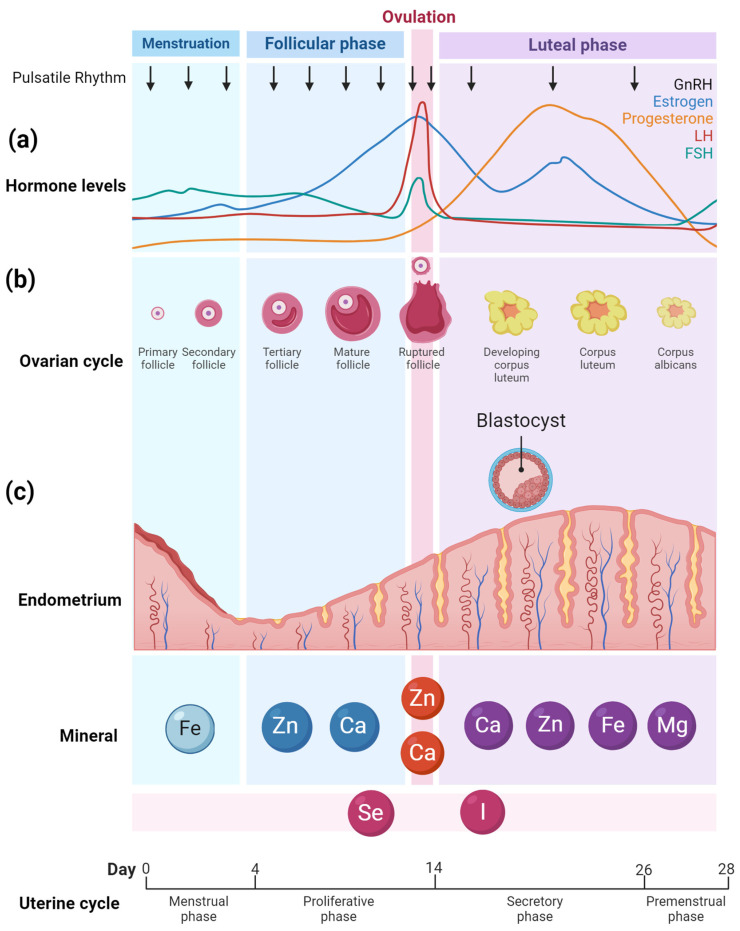
Minerals and their influence on hormonal regulation, ovarian function, and endometrium throughout the menstrual cycle. The graph (**a**) illustrates hormone fluctuations during the menstrual cycle, the sequence of images (**b**) depicts the progression of ovarian follicle development, and the illustration (**c**) shows the corresponding changes in the endometrium’s thickness. In subfigure a, the different colors represent the varying levels of specific hormones over the course of the menstrual cycle: black for GnRH, blue for estrogen, orange for progesterone, red for luteinizing hormone (LH), and green for follicle-stimulating hormone (FSH), each peaking at different times to regulate the cycle.

Human reproduction is regulated by hormones, guiding processes from the onset of menstrual cycles during puberty to the complexity of ovulation, implantation, and gestation [14]. At the cycle’s outset, declining estrogen and progesterone levels result in the elimination of the endometrial lining. As the follicular phase progresses, Follicle-Stimulating Hormone (FSH) promotes ovarian follicle growth, leading to an estrogen increase which suppresses FSH production. A Luteinizing Hormone (LH) increase, stimulated by peak estrogen levels, marks ovulation and the release of a mature egg. Post-ovulation, in the luteal phase, the transformed follicle—now the corpus luteum [15]—secretes progesterone and some estrogen, readying the endometrial lining for potential implantation [16]. FSH and LH are essential in this reproductive process. They regulate follicle maturation and ovulation, while estrogen and progesterone, produced by the follicles and corpus luteum, prime the endometrium for optimized conditions for a fertilized egg [17] (Figure 1a). While insulin is predominantly recognized for its role in regulating glucose metabolism, it also plays a crucial role in reproductive processes. 

Iron (Fe) is essential for the menstrual cycle [18,19,20], with deficiencies causing hormonal imbalances [21,22,23,24,25] and anemia that affect blood flow to the ovaries and ovulation regularity [26,27,28]. During the follicular phase and ovulation, selenium (Se), calcium (Ca), and zinc (Zn) play pivotal roles. Ca is integral to gonadotropin-releasing hormone (GnRH) regulation [29,30], oocyte activation, and zygotic development [31,32], and it also modulates second messengers vital for sperm fusion [33,34,35]. During the first half of the menstrual cycle, known as the follicular phase, increased GnRH secretion stimulates the release of FSH and LH, which are crucial for follicle development and preparing the body for ovulation; in the second half, the luteal phase, GnRH secretion is reduced as rising progesterone levels from the corpus luteum exert negative feedback on the hypothalamus and pituitary gland [36]. Zn aids hormone synthesis [37] by influencing LH, FSH [38], and steroid synthesis [39,40], and, along with Se [41,42], safeguards the oocyte from reactive oxygen species (ROS) damage [43,44]. In the luteal phase, Ca [45,46], Zn [47,48], Fe [49,50,51,52,53], and magnesium (Mg) [54,55] collectively support endometrial function [56]. Ca, deposited at the embryo implantation site, controls endometrial receptivity [45,46]. Deficiencies in Fe [49,50,51,52,53] and zinc can impair this receptivity and hinder embryo implantation [47,48]. Mg, by relaxing smooth muscle [54], may influence retrograde menstruation [54,55] and reduce vascular endothelial growth factor, offering potential benefits for conditions like endometriosis [57,58,59]. Both iodine (I) and Se [60,61], due to their ties with thyroid hormones, are vital throughout the menstrual cycle [62]. Their deficiencies can disrupt hormonal balance [63], endometrial health [64], follicle development [65], and overall fertility [65,66]. Adapted from “Ovarian hormones throughout the menstrual cycle”, by BioRender.com (accessed on 27 March 2024).

Elevated levels of insulin can prompt the ovaries to produce increased androgens, such as testosterone, potentially interfering with processes governed by estrogen and progesterone [67,68]. Thus, balanced insulin levels are pivotal for both metabolic and reproductive efficiency [69]. Imbalances in insulin levels, as observed in conditions like polycystic ovary syndrome (PCOS), can disrupt hormonal pathways [14,70]. PCOS is a multifactorial endocrine disorder characterized by ovarian dysfunction, hyperandrogenism, and metabolic disturbances, with implications for reproductive, metabolic, and cardiovascular health [71].

### 2.2. Influence of Minerals on Hormonal Regulation

#### 2.2.1. Zinc

Zinc (Zn) plays a multifaceted role in our body, particularly in hormonal activities. Not only is it vital for insulin metabolism [72], but also in testosterone synthesis [39,40,73]. Zinc finger proteins are involved in the genetic expression of steroid hormone receptors. Therefore, zinc plays a pivotal role in the expression of various hormonal activities, including the action of the metabolism of androgen hormones, estrogen, and progesterone [74,75,76]. 

For women, maintaining a balance of sex hormones is crucial: Any imbalances could result in reproductive issues [77,78,79]. The risk of preterm delivery was increased with low zinc intake (< or =6 mg/day) [77]. Testosterone, synthesized in the ovaries’ theca cells, is essential for follicle maturation [40]. A balance between the different sex hormones is necessary for the proper functioning of the menstrual cycle and follicle maturation [80,81]. Testosterone plays a key role in the regulation of female libido. A deficiency of testosterone can lead to reduced sexual desire [80]. Additionally, zinc interacts with steroid hormones [82] and is imperative for the proper functioning of estrogen receptors [83,84], which play a central role in the menstrual cycle [85,86] and overall female reproductive health [40,87,88]. Research has also underscored the involvement of zinc in follicular development [89,90,91,92,93] and ovulation [94,95,96]. Consequently, maintaining optimal zinc levels may be crucial for women aiming to optimize their fertility, especially at the time of the follicular phase and ovulation (Figure 1a) [75,97].

Throughout the menstrual cycle, serum zinc concentrations vary. According to a study by Michos C. et al., plasma zinc levels changed significantly between all times of the menstrual cycle. The highest levels were observed during ovulation and the lowest at the time of menstruation [76]. However, there is no clear relationship between serum zinc concentrations and infertility, but it highlights the importance of zinc for proper menstrual cycle function [37,98,99,100].

A zinc deficiency (<56 μg/dL) [78] can have adverse effects on women’s reproductive health. It might lead to issues like the altered synthesis or secretion of FSH and LH, irregular ovarian development, menstrual cycle disturbances, asynchronous uterine contractions, and even conditions like pre-eclampsia [37,38].

Research also hints at a potential link between zinc deficiency and PCOS pathogenesis, especially concerning insulin resistance [101] and testosterone levels [101,102]. Insulin resistance is a pronounced characteristic in many women with PCOS, and mean serum zinc levels were also significantly lower in PCOS women [101,103]. Investigations have demonstrated that zinc can enhance insulin sensitivity by acting as a cofactor for several enzymes engaged in carbohydrate metabolism [104]. Furthermore, zinc supplementation with 30 mg or 40 mg of zinc sulfate can optimize insulin secretion and its receptor binding [105], leading to improved glucose uptake and utilization [106]. 

#### 2.2.2. Selenium

Selenium is essential for the production of selenoproteins, which are crucial for the conversion of the thyroid hormone thyroxine (T4) into its active form, triiodothyronine (T3) [60,63]. The proper transformation of T4 to T3 is central for regulating metabolic rate and numerous cellular processes within the body [61,64,107,108,109]. Proper thyroid function is crucial for a balanced hormonal environment in the female reproductive system [110]. Conditions like hyperthyroidism or hypothyroidism can disrupt the menstrual cycle [111,112], increase follicular atresia [113], lead to anovulation [114], and lead to impaired fertility [115,116,117]. Thyroid hormones both directly and indirectly modulate other essential reproductive hormones, such as progesterone and estrogen [63,115,118]. Ensuring optimal selenium levels might help manage the impact and severity of thyroid-related conditions, thereby influencing female reproductive health. 

For optimal hormone regulation and consistent menstruation without anovulation, selenium is especially vital during the follicular phase, around ovulation, and throughout menstruation. Furthermore, the importance of selenium is not limited to the earlier phases, as it also has a crucial role in the luteal phase. Research conducted by Zagrodzki P. et al. [119,120] explored the interplay between selenium status, secretion of sex hormones, and thyroid metabolism in both adolescent girls and adult women. Their findings underscore a clear association between selenium levels and the female reproductive system, with particular emphasis on how selenium influences thyroid physiology in adolescent girls and young women during the luteal phase [119,120].

#### 2.2.3. Iodine

Iodine is predominantly recognized for its pivotal role in thyroid function and plays a crucial role in the synthesis of thyroid hormones [121]. As mentioned above, these hormones interplay with reproductive hormones, such as progesterone and estrogen [115]. Thus, an imbalance due to either a deficiency or an excess of iodine can set off a cascade of effects, leading to disruptions in reproductive hormones. Such hormonal disruptions can hinder ovulation and compromise overall reproductive health, highlighting the subtle yet significant influence of iodine on fertility dynamics [62,66,121,122,123,124].

Iodine deficiency is one of the most common causes of hypothyroidism worldwide [125]. Iodine deficiency (iodine–creatinine ratios below 50 μg/g) in women is directly related to infertility, as reported by Mills and colleagues [123]. Hypothyroidism due to iodine deficiency in women results in significant reproductive changes, including anovulation [65] and decreased fertility [66], and when pregnancy occurs, gestational hypertension, stillbirths, and congenital anomalies, as well as increased perinatal mortality, may be observed [126,127,128]. A decline in thyroid function is associated with diminished blood-binding capacity for sex hormone-binding globulin (SHBG) [129]. SHBG plays a pivotal role in sequestering and transporting gonadal hormones in the circulatory system [130]. As a corollary of this diminished binding capacity, there is a noted decrease in the total concentrations of testosterone and estradiol, a primary estrogen [131]. Concurrently, there is an uptick in the fraction of these hormones that remain unbound to proteins [132]. Additionally, hypothyroidism can attenuate the response to LH [129], imperative for ovulation [133]. This attenuation can subsequently stimulate the secretion of thyrotropin-releasing hormone (TRH) [134]. An elevated concentration of this hormone can induce a surge in serum prolactin levels [135]. Elevated prolactin can disturb the consistent secretion of gonadotropin-releasing hormone (GnRH) [118], a critical regulator for the onset of the menstrual cycle and ovulation [136,137]. Such perturbations can manifest as ovulatory dysfunctions [138,139].

Due to the direct relationship between iodine and thyroid physiology, adequate iodine intake, just like selenium, must be maintained throughout the menstrual cycle (Figure 1a).

#### 2.2.4. Iron 

Iron is an essential component of hemoglobin, the protein in red blood cells that carries oxygen throughout the body [140]. Adequate oxygen transport is necessary for many bodily functions, including the function of the ovaries and uterus [18,140]. Beyond its function in oxygen transport, iron is essential to the operation of certain enzymes. These enzymes affect multiple biochemical pathways, including those related to hormone synthesis and regulation [18,19,20]; estrogen and iron metabolism in particular are closely related [141,142]. For instance, the Cytochrome-P450 enzymes, which process steroid hormones like estrogen and progesterone, significantly depend on iron [143]. Similarly, iron is indispensable for the enzymes Prolyl Hydroxylase [144] and Lysyl Hydroxylase [145] involved in collagen synthesis [146], thereby influencing the health of the connective tissue in reproductive organs [147]. As a consequence, an iron deficiency, often manifested as anemia, can lead to disturbances in menstrual cycles. Hormonal imbalances from such deficiencies can not only affect the regularity of the menstrual cycle but also reduce fertility potential [21,22,23,24,25].

#### 2.2.5. Calcium

Calcium is renowned primarily for its role in bone health [148,149], but it also plays a significant role in the secretion and action of hormones [150], particularly in the synthesis and secretion of neurotransmitters [151]. These neurotransmitters influence the release of GnRH. The activity of GnRH neurons, especially their “burst-firing” mode, is affected by calcium ions [152]. This burst-firing activity is closely linked to the release of GnRH, a key hormone that controls the female menstrual cycle [153,154]. By stimulating the anterior pituitary gland, GnRH prompts the release of LH and FSH, which subsequently induce the ovaries to produce and release estrogen and progesterone. Disruptions in GnRH release can affect ovulation and thereby influence female fertility [29,30,151,152,155,156]. Additionally, calcium is involved in the release of insulin from the pancreas, highlighting its indirect role in metabolic pathways associated with hormonal regulation [14,157,158,159,160]. Calcium plays an important role, particularly during the ovulatory phase, as it influences the release of gonadotropin-releasing hormone (GnRH), which controls ovulation by stimulating the release of LH.

#### 2.2.6. Magnesium

Magnesium is an essential mineral that plays a central role in hormonal regulation in the body, thereby also influencing female fertility [161]. It serves as a cofactor for a variety of enzymatic reactions, including those directly linked to the production and function of reproductive hormones. For instance, magnesium is involved in the activity of enzymes such as aromatase [162,163], responsible for converting androgens into estrogens [164].

Disruptions in these hormonal harmonies, as seen in conditions like PCOS, can spell challenges for fertility [165,166,167,168,169,170,171,172,173,174,175]. Through these mechanisms, adequate magnesium levels contribute to optimizing insulin sensitivity and stabilizing glucose metabolism. Research indicates that women with PCOS often exhibit lower serum magnesium levels compared to those without the condition [165,166,167,168,169,170,171,173,174]. Consequently, magnesium supplementation may represent a promising therapeutic avenue for PCOS patients to ameliorate insulin metabolism and reduce the risk of associated comorbidities [161,176,177,178,179,180]. While current research is not clear about the specific phase of the menstrual cycle where magnesium has the strongest influence on hormones, it is consistently emphasized that maintaining adequate magnesium levels throughout the cycle is crucial.

## 3. Ovarian Function and Ovulation

### 3.1. Overview of Ovarian Function

The ovaries, paired almond-sized organs located on each side of the uterus, play a central role in female reproductive health. Their primary functions include the production of oocytes for fertilization and the synthesis of key hormones, primarily estrogen and progesterone, which regulate menstrual cycles and support pregnancy [102,181,182,183,184,185,186,187].

The life of an oocyte begins with a primary follicle growing into a mature follicle (Figure 1b) and ends in the fetal period. Female fetuses have approximately 6–7 million potential follicles; however, by puberty, only about 400,000 remain [188,189]. Each menstrual cycle then witnesses a cohort of these follicles initiating development, though usually only one reaches full maturity and undergoes ovulation [190].

### 3.2. Influence of Minerals on Ovulation

#### 3.2.1. Calcium

Calcium plays an important role in ovarian function and the progression of the female reproductive system [191,192,193]. Within the ovary, calcium influences follicular development and oocyte maturation (as in Figure 1b) [194,195,196]. It is instrumental in the intracellular signaling pathways that govern ovulation, facilitating the release of the oocyte from the follicle. Some of these crucial intracellular signaling pathways include the Store-Operated Calcium Entry (SOCE) [197], the Calcium/Calmodulin-dependent Protein Kinase II (CaMKII) pathway [198], and the Phospholipase C (PLC) pathway [199]. Additionally, calcium is indispensable during fertilization, participating in oocyte activation and the initiation of zygotic development [31,32]. Specifically, during these stages, calcium ions modulate the release of vital secondary messengers, including inositol trisphosphate (IP3) [33], cyclic adenosine monophosphate (cAMP) [34], and diacylglycerol (DAG) [35], which are essential for oocyte activation and its fusion with the spermatozoon [31,32]. In the early stages of embryonic development, a balanced calcium concentration is crucial for proper cellular division and the implantation of the embryo into the uterine lining [31,200,201,202,203]. Consequently, an imbalance in calcium levels, whether due to a deficiency [204,205] or excess [206], can compromise fertility and reduce the likelihood of a successful pregnancy [207]. Thus, for women aiming to conceive, it is crucial to maintain optimal calcium homeostasis to ensure the most conducive environment for fertilization and embryonic progression. In summary, calcium is particularly important during the phases of follicular development, egg maturation, ovulation, and fertilization in the female reproductive cycle.

#### 3.2.2. Zinc

Zinc plays an integral role in oocyte maturation, quality, and functionality, serving as a pivotal element in various cellular and metabolic pathways crucial for the oocyte’s proper development and maturation [208,209]. It acts as a crucial cofactor for enzymes like DNA polymerase [210], ribonucleotide reductase [211], and thymidylate synthase [212], vital for DNA synthesis and repair [213,214]. Moreover, zinc influences cell cycle regulators such as zinc finger proteins [215] and the p53 protein [216], contributing to cell cycle regulation and cellular protection [37,93,217]. Numerous scientific investigations have highlighted that zinc deficiency can significantly perturb follicular development, a critical process wherein the oocyte matures within a protective sac of granulosa cells [88,94,218]. Disruptions in this complicated process, due to a zinc deficiency (Tian and Diaz 2013), can lead to ovulation disorders, which are characterized by impaired follicle rupture or impaired egg release [219]. Consequently, this diminishes the potential for successful fertilization and implantation [220,221,222].

Furthermore, zinc insufficiency can compromise oocyte quality [223], further attenuating its capacity for fertilization and subsequent embryonic development [91,224,225,226,227] potential for oocyte growth [228]. A deficiency in zinc triggers apoptosis that restricts the proliferation of cumulus cells [229]. These cells are vital for oocyte maturation, aiding in cytoplasmic maturation and growth by synthesizing glutathione (GSH) and delivering it to the oocytes. Thus, the suboptimal growth and development of cumulus cells detrimentally affect oocyte maturation and quality [230]. 

#### 3.2.3. Iron 

Iron, an indispensable trace element in the human body, plays a pivotal role in various physiological processes, including oxygen transport via hemoglobin in the bloodstream [231]. The ovaries, as specialized reproductive organs, rely heavily on a consistent and adequate oxygen supply to ensure the proper maturation of oocytes, which are crucial for successful fertilization and subsequent embryo development [232,233,234,235,236]. Iron’s involvement in DNA synthesis is important for the rapid cellular division observed during oocyte maturation and early embryonic stages [237]. Furthermore, iron is essential for mitochondrial function, ensuring that cells, including those in the ovaries, have the necessary energy for their intricate processes [235,238]. A deficiency in iron can lead to anemia, which may compromise the blood flow to the ovaries, potentially affecting the quality of oocytes and the regularity of ovulation [26,27,28]. This can have downstream effects on overall fertility, making the maintenance of optimal iron levels paramount for women wishing to conceive. A case–control study by Holzer et al. [239] in 2023 demonstrated an association between low ferritin levels—a substance with which cells can store iron—and unexplained infertility [239]. Thus, ensuring appropriate iron levels is not only vital for general health but is also intricately linked to female reproductive success. There has also been a significant association between iron overdose and lower egg counts noted in assisted reproductive technologies (ART) studies [53,240].

Two studies, one from 2022 [241] and one from 2023 [242], with 6551 participants suggest an association between changes in iron metabolism, particularly elevated ferritin levels, and ovarian endometriosis. Endometriosis is a chronic condition characterized by the presence of endometrial-like tissue outside the uterine cavity [243,244] leading to a microenvironment established by endometriotic lesions, which is dominated by inflammation and oxidative processes [245]. Ferritin and iron levels were found to be higher in cyst fluid than in serum, suggesting local iron accumulation [246]. The risk for endometriosis appears to increase with ferritin and transferrin levels up to a certain threshold, after which it stagnates [241,242,246]. However, further research is needed to clarify the exact role of iron metabolism in endometriosis.

## 4. Oxidative Stress and Fertility

### 4.1. Overview of Oxidative Stress and Its Influence on Fertility

Oxidative stress refers to an imbalance between the production of free radicals and the body’s ability to counteract or detoxify their harmful effects using antioxidants [247,248]. Free radicals, primarily reactive oxygen species (ROS), can cause damage to cellular structures, including DNA, proteins, and lipids [247,248].

**Figure 2:**
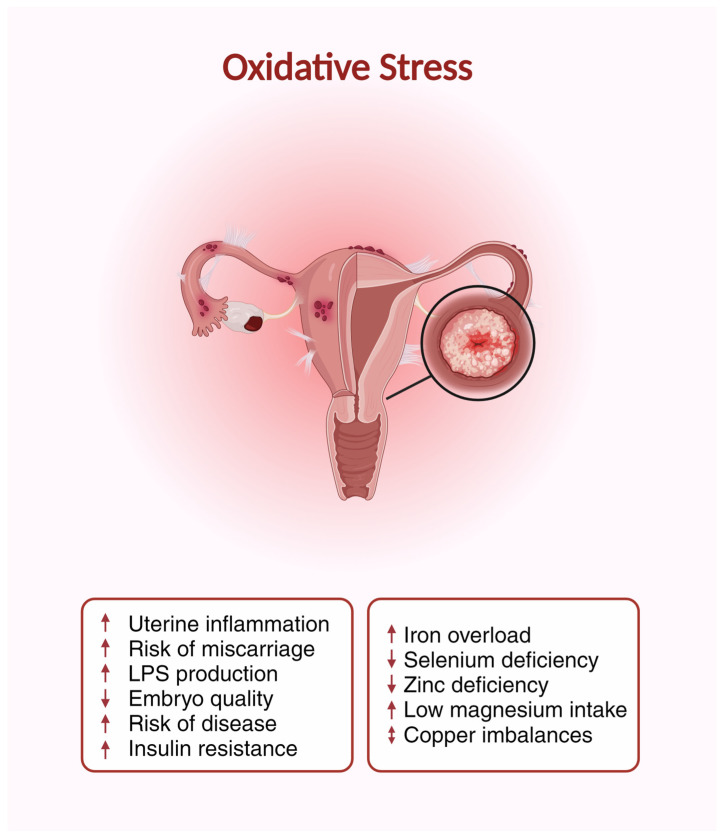
Oxidative Stress on the Uterus.

In the context of reproductive health, oxidative stress has been shown to play a pivotal role. Elevated ROS levels can impact the quality of both sperm [249] and oocytes [250], compromising their function and potentially reducing the chances of fertilization [251]. For instance, excessive ROS can cause DNA fragmentation in spermatozoa [252], diminishing its fertilizing potential. Likewise, oxidative stress in the ovarian microenvironment can impact the maturation, quality, and function of oocytes [183,251,252]. Moreover, post-fertilization, excessive ROS can hinder embryo development and implantation [253]. Diseases that cause infertility, such as endometriosis [254] and PCOS [255], have also been shown to have increased ROS levels. Minerals play a crucial role in the body’s defense mechanism against oxidative stress [256] (Figure 2). Their participation in enzymatic antioxidant systems aids in neutralizing the adverse effects of ROS [257].

Oxidative stress plays a central role in impairing female fertility [183]. Direct effects include increased uterine inflammation [258], increased risk of miscarriage [259], decreased embryo quality [43,183], increased risk of disease associated with reproduction [245,258,260], and growing insulin resistance that potentially impairs fertility [261]. Iron overload [262], deficiency of selenium [263], zinc deficiency [264], low magnesium intake [265], and imbalance of copper [266] in the body promote oxidative stress and, thus, have indirect effects on fertility. LPS (lipopolysaccharide) is a component of the outer membrane of Gram-negative bacteria that can promote oxidative stress, which impairs female fertility by increasing inflammation and damaging egg cells [267]. This illustration was created using BioRender.

### 4.2. Influence of Minerals on Oxidative Stress

#### 4.2.1. Zinc

Zinc has remarkable antioxidant properties that make it an essential component in protecting the body from oxidative stress [106,264,268]. Zinc serves as a cofactor for various antioxidant enzymes, including superoxide dismutase (SOD) [268]. SOD facilitates the conversion of superoxide radicals into less harmful molecules, thus mitigating oxidative damage. Research has emphasized the importance of zinc in maintaining the optimal activity of SOD, offering protection against oxidative stress-induced damage in the reproductive system [37,209,269,270,271]. In the context of female fertility, zinc’s antioxidative function plays a pivotal role. It safeguards the oocytes, which are especially vulnerable to ROS-induced damage during maturation, thereby enhancing the likelihood of successful fertilization [99,272,273]. Concurrently, zinc facilitates the ovulation process by maintaining an ROS balance, promoting a consistent ovulatory cycle [43,219]. Post-fertilization, zinc serves a protective role for the developing embryo against ROS-induced harm, augmenting the prospects for successful implantation and development [44,274,275,276,277]. Therefore, an optimal zinc status can significantly augment a woman’s ability to produce healthy oocytes, ovulate successfully, and ultimately experience a successful pregnancy [209,221,278]. A deficiency in zinc can exacerbate health complications across various diseases, compromise immune functionality [279], and augment oxidative stress [280]. Despite limited research linking zinc to endometriosis, evidence suggests diminished zinc levels in afflicted women. This is particularly evident in the follicular fluid of the ovaries when compared to women with fallopian tube-related infertility [48,281]. Furthermore, zinc possesses anti-inflammatory attributes that might modulate the inflammatory processes so commonly observed in endometriosis, potentially increasing infertility [59,282,283]. However, proper dosing is essential since excessive zinc, though essential for myriad physiological processes, can exhibit pro-oxidative effects [279,284,285,286].

#### 4.2.2. Selenium

While the association between selenium status and reproductive function is well-established in males [287,288], its elucidation in females remains an active area of investigation. Selenium plays an important role in protecting against oxidative damage, especially concerning fertility [41,263]. Optimal selenium concentrations are associated with enhanced fertility outcomes, attributable to the mitigation of oxidative stress in reproductive tissues [289,290,291].

Selenium’s antioxidative properties are integral to reproductive health [42]. Selenium is crucial to the function of the antioxidant enzyme glutathione peroxidase (GPx). GPx neutralizes hydrogen peroxide, a potent ROS, by converting it to water, preventing lipid peroxidation and potential damage to cellular membranes [292,293]. Oxidative stress, stemming from an excessive presence of free radicals, can adversely affect the ovulatory process [42,248,294]. This encompasses damage to ovarian follicles, compromised oocyte quality, and perturbations in the intricate hormonal equilibrium required for ovulation [295,296]. Selenium acts as a cofactor for a gamut of antioxidant enzymes, notably glutathione peroxidases, which counteract free radicals, attenuating the repercussions of oxidative stress on the ovaries [297,298,299,300]. Numerous studies emphasize the importance of maintaining optimal selenium levels to protect the ovaries from oxidative damage, thus ensuring a stable and efficient ovulation process [298,301]. In patients with endometriosis, a combined intake of vitamin E, C, selenium, and zinc was inversely correlated with disease severity [302]. A lower consumption of these antioxidants was associated with an increased intensity of the disease, suggesting a potential link between disease progression and the state of lipid peroxidation [59,302,303].

#### 4.2.3. Copper 

Copper is also essential in the body’s defense against oxidative stress, primarily as a cofactor for the enzyme Superoxide Dismutase (Cu, Zn-SOD) [266]. Beyond this, copper also modulates other antioxidative systems and can influence signal transduction processes and gene expression [304,305,306,307]. Pertaining to female reproduction, copper’s antioxidative properties serve to protect oocytes from oxidative stress, which is vital for their integrity and function [307,308]. Additionally, copper supports endothelial function, crucial for optimal blood flow to the uterus and ovaries, thus positively influencing fertility [309,310,311,312,313]. Notably, copper can also manifest pro-oxidative properties when present in excessive amounts [313]. 

#### 4.2.4. Iron 

Iron, an essential trace element within the human body, plays a dual role in relation to oxidative stress and reproductive health. On one hand, it is crucial for oxygen transport and DNA synthesis. On the other hand, an excess of circulating non-transferrin-bound iron can increase the production of ROS, leading to oxidative stress [262,314,315]. 

However, conditions such as hereditary hemochromatosis, a genetic disorder, can lead to hyper-absorption and excessive deposition of iron within the body [316,317]. Similarly, indiscriminate iron supplementation without appropriate medical indication can elevate oxidative stress risk [318].

Additionally, the susceptibility of oocytes to oxidative stress intensifies with age, a phenomenon known as ovarian aging [319,320,321]. In this context, iron can paradoxically play both protective and detrimental roles [23,321,322]. While adequate iron levels, supplemented in cases of deficiency, can increase oocyte quality [22], an excess can diminish it, augmenting the risk of compromised fertility [25,53].

Inflammatory conditions can perturb the balance of iron, amplifying oxidative stress. This disturbance, coupled with the direct detrimental effects of inflammation, can significantly compromise oocyte quality, thereby negatively influencing fertility [24,102,236,323].

Thus, maintaining an appropriate balance of iron within the body is paramount to ensuring optimal reproductive health and fertility in women [22,324].

#### 4.2.5. Magnesium 

Magnesium serves as a cofactor for over 600 enzymatic reactions [325], including those involved in the repair of DNA damage [326] induced by oxidative stress [327]. It is also essential for the proper functioning of glutathione, a primary antioxidant in the body [328,329]. A sufficient magnesium status can mitigate the production of free radicals that pose harm to cellular structure [330,331,332]. Conversely, magnesium deficiency has been demonstrated to exacerbate oxidative stress [333]. The antioxidative properties of magnesium may indirectly bolster fertility [334] by safeguarding against oxidative stress, which can detrimentally affect reproductive health [161,335,336]. Through its antioxidative attributes [327,337], magnesium can preserve oocyte quality [251], modulate the ovulatory process [251,338], and contribute to the healthy functioning of the endometrium (Figure 2) [115,183,325,337,339,340]. 

Based on this information, magnesium plays a crucial role during the follicular phase, concerning the maturation and quality of oocytes and the ovulation process. Additionally, it is significant during the luteal phase in relation to the healthy functioning of the endometrium.

#### 4.2.6. Manganese 

Manganese, an essential trace element, holds a distinguished role as an antioxidant, primarily as a cofactor for the enzyme manganese superoxide dismutase (Mn-SOD), which is pivotal in protecting cellular structures against oxidative stress [341,342]. As mentioned before, in the context of female fertility, oxidative stress can adversely affect oocyte quality and function. It may also disrupt hormonal equilibrium and the reproductive cycle [343]. Therefore, manganese, through its antioxidative attributes, may play a protective role in female reproductive health and enhance female fertility [344,345]. However, manganese, while an antioxidant at physiological levels, can contribute to oxidative stress in surfeit [346]. Consequences of such pro-oxidative states range from the degradation of oocyte quality to perturbation in ovarian function [347,348,349]. 

## 5. Endometrium and Embryo Implantation

### 5.1. Overview of Implantation and Endometrium in Fertility

The uterus, enveloped by its specialized endometrial lining [350], is paramount for embryo implantation and subsequent gestation [351]. This muscular organ, in its entirety, goes through considerable dynamic changes across the menstrual cycle [352]. The endometrium, its inner lining, undergoes thickening, secretion, and shedding, all intricately timed and regulated by hormones [353,354]. In a successful conception cycle, the fertilized embryo finds a receptive endometrium, initiates implantation, and sets the stage for continued gestation [353,355].

Implantation is the process when the blastocyst, having completed multiple divisions after fertilization, attaches to the receptive endometrial lining [356] (Figure 1c). This process commences roughly a week after ovulation and is a highly coordinated interplay between the embryo and the maternal endometrium [357].

Upon reaching the uterine cavity, the blastocyst emerges from its protective shell, the zona pellucida, in a process termed ‘hatching’ [358]. The trophectoderm cells of the hatching blastocyst then initiate adhesion by recognizing specific molecules on the luminal surface of the endometrium. Subsequently, the blastocyst delves into the endometrial stroma, facilitated by the enzymes it secretes [359]. At the same time, the endometrium undergoes a transformation into a decidua, creating a nutrient-rich environment that nurtures the swiftly dividing embryo [360].

### 5.2. Influence of Minerals on Implantation and Endometrium

#### 5.2.1. Iron

An iron deficiency can entail significant challenges for both the mother and the budding fetus [361,362]. Iron was investigated in relation to the endometrium. A study by Rodriguez-Diaz et al., 2023 has revealed that a deficiency in iron may impact endometrial conditions and its receptiveness, potentially decreasing the chances of successful embryo implantation [53]. Additional research also underscores the significance of iron balance within both the endometrium and the embryo, suggesting this balance plays a crucial role in the endometrium’s receptivity to an embryo and the subsequent implantation process [49,50,51,52,53]. There is promising research regarding Fractalkine (FKN) [363,364]. It has been found that FKN mitigates the negative impacts of iron deficiency on the receptivity-related genes and proteins in human endometrial carcinoma cells HEC-1A [363].

Overall, these results suggest that iron may play an important role in the development of endometrial receptivity and embryo implantation (Figure 1c). Therefore, a balanced iron metabolism could be crucial for a successful pregnancy.

#### 5.2.2. Magnesium

Studies have demonstrated that in women with endometriosis, the fallopian tubes contract irregularly and spasmodically. Magnesium is known to relax smooth muscle, potentially influencing retrograde menstruation, which is considered a primary cause of endometriosis [54,55]. A study on rats by Hosgorler et al. suggests that magnesium can decrease the levels of vascular endothelial growth factor (VEGF) in uterine tissue, which may be beneficial in the treatment of gynecological conditions like endometriosis [57,58,59]. However, human studies on the use of magnesium in endometriosis are limited, necessitating further research.

#### 5.2.3. Zinc

Zinc extends its physiological influence far beyond DNA synthesis [365]. In the intricate landscape of the endometrial lining, zinc emerges as a key regulator, governing cellular proliferation and differentiation [56,366]. These processes are essential for the cyclical endometrial shifts that form the foundation of menstrual cycles, with the maturation and shedding of the endometrial layer being tightly regulated events [37,47,367]. 

A study by Onuma et al., 2023 suggests that zinc deficiency may play a role in the development of endometrial cysts, a type of ovarian cancer [48]. Patients with endometrial cysts exhibited lower serum zinc levels compared to those with benign non-endometrial cysts. In experimental assays, zinc depletion from endometrial epithelial cells led to enhanced cell proliferation, indicating that zinc may have a potential inhibitory role in this growth [48]. Additionally, it was observed that zinc deficiency influenced specific gene expression alterations, which were neutralized upon the reintroduction of zinc [48,281,368]. These findings propose that zinc supplementation might serve as a potential therapeutic strategy to impede the development of such cysts.

#### 5.2.4. Calcium

During pregnancy, the amount of calcium in the uterine endometrial tissue increases, and calcium deposits are made at the site where the embryo implants in the uterus. These are critical steps for the onset of pregnancy [45,369,370]. The hormone estrogen has been identified as a factor that increases the uptake of calcium in the body and influences the activity of specific genes [371,372] in the endometrium that are crucial for the initiation of pregnancy [45,369,370]. Among the significant genes related to uterine function and development is the Homeobox A10 (HOXA10) gene [373]. It plays a pivotal role in uterine development and function [374,375]. Alterations in the expression of HOXA10 have been associated with implantation failure [376]. Furthermore, the Leukemia Inhibitory Factor (LIF) is another crucial molecule linked to embryo development [377]. Overall, these results indicate that an adequate calcium level in the body is a requirement for the successful implantation of the embryo and the initiation of pregnancy [45,203,370,378]. 

The results of another study by Zhang et al. demonstrate that calcium, in the form of large-conductance calcium-activated potassium channels (BK(Ca) channels), is expressed in the human endometrium and plays a significant role in regulating endometrial receptivity and embryo implantation [46]. The expression of these channels varies throughout the menstrual cycle and influences the attachment of embryos to the endometrial lining, as well as the expression of factors crucial for endometrial receptivity [379]. Additionally, BK(Ca) channels regulate the activity of NF-κB, a transcription factor involved in inflammatory processes and cell proliferation, while also affecting calcium homeostasis in endometrial cells. These findings suggest that calcium serves as a crucial regulator of endometrial function, playing a pivotal role in successful embryo implantation and pregnancy. Disorders in calcium metabolism may consequently impact fertility and reproduction [46].

#### 5.2.5. Iodine

While limited research has been conducted in humans, studies using animal models have provided insights into the relationship between iodine and fertility, particularly its effects on the endometrium. In cows with unexplained infertility (UI), treatment involving uterine infusions of Lugol’s iodine has been found to improve fertility. This improvement has been attributed to the bactericidal properties of iodine, which may aid in the restoration of damaged endometrial tissue. Other proposed mechanisms include alterations in uterine pH and enhanced uterine blood flow [380]. Similarly, the intrauterine infusion of Polyvinylpyrrolidone-Iodine (PVP-I) in dairy cows can induce transient uterine inflammation while promoting the regeneration of endometrial epithelial cells and improving fertility. This suggests that iodine, present in PVP-I, may potentially play a role in enhancing uterine lining and embryo implantation [381,382]. Rats treated with Lipiodol, a substance containing iodine, exhibited changes in the phenotype of dendritic cells in the endometrium [383]. These cells play a role in regulating the establishment and maintenance of implanted embryos. Iodine in Lipiodol may be responsible for these immunological changes, potentially enhancing embryo implantation [124]. The results of a randomized controlled trial study suggest that Lipiodol may be effective as a fertility treatment for women with unexplained infertility and endometriosis-related infertility [384]. The exact mechanism by which Lipiodol affects fertility is not yet fully understood, but it is thought that it may improve the receptivity of the endometrium to embryo implantation. While smaller doses of iodine appeared beneficial in creating a favorable uterine environment for reproduction in rats, the infusion of a large iodine dose into the uteri of mares resulted in severe tissue damage. This suggests that high doses of iodine can be toxic [124,385]. Adverse effects of maternal iodine excess were also shown by Sihan Wang et al. in 2023, and a negative effect on the neurological development and physical growth of infants was also shown during the early stages of pregnancy. In the study, it was shown that maternal serum iodine concentrations above 92 µg/L in the first trimester had adverse effects on infants’ development, while in the third trimester, levels above 92 µg/L positively influenced infants’ height. Suitable serum iodine concentration values in the first trimester ranged from 40 to 92 µg/L [386]. However, it is also noted that maternal iodine excess during the third trimester may have a positive impact on infant length growth [127,386,387]. Additionally, in a study of 501 women with moderate to severe iodine deficiency, pregnancy was delayed, and the likelihood of conception in each cycle decreased by 46% compared to non-iodine-deficient women [4,123,388]. 

## 6. Conclusions

In conclusion, based on this comprehensive review of the role of minerals in female fertility, it has become abundantly clear that each mineral examined—zinc, magnesium, calcium, iodine, selenium, iron, copper, and manganese—plays a significant role in the biological processes that underpin reproductive health. The interactions of these minerals with key determinants of fertility such as hormonal regulation, ovarian function, ovulation, oxidative stress, and endometrial implantation are complex and critical (Table 1).

Ensuring the optimal health of the female reproductive organs requires the careful regulation of mineral homeostasis, as zinc is essential for the modulation of insulin metabolism, steroidogenesis, and the control of ovulation cycles. It also protects the embryo from oxidative damage and supports endometrial cell proliferation. A zinc deficiency can manifest itself in impaired folliculogenesis [88,94,276] and menstrual irregularities [37], while an excess of zinc can increase pro-oxidative conditions and thus impair fertility [253,286].

Magnesium serves as a central cofactor in estrogenic metabolic pathways [389] and in glucose homeostasis [390], which is particularly important in people with polycystic ovary syndrome (PCOS) [391]. Hypomagnesemia can increase oxidative stress and impair oocyte viability [392] and endometrial functionality [183]. Normomagnesemia is generally maintained by renal regulatory mechanisms, so hypermagnesemia is less likely to be a problem [393].

Calcium, an important regulator of gonadotropin-releasing hormone (GnRH) secretion [394], is critical to the mechanisms of ovulation and embryo implantation [395]. Imbalances in calcium homeostasis—be it hypocalcemia or hypercalcemia—can hinder gametic [45,46] and zygotic development [31,32], emphasizing the need for balanced calcium intake during the follicular and ovulatory phases [191,395].

Iodine’s critical involvement in thyroxine synthesis and the consequent impact on reproductive hormones implicates its deficiency in anovulation and cyclical disturbances [65,66]. Excess iodine, particularly during the early stages of pregnancy [383,384,386], is associated with adverse developmental outcomes, necessitating prudent iodine management across the reproductive spectrum. 

Selenium, embedded within the selenoprotein-dependent pathways of thyroid hormone conversion [60,61], is essential for ovarian protection and ovulatory integrity [41,42]. Selenium deficiency may lead to menstrual and ovulatory disruptions, whereas its excess can lead to toxicity [41,42]. 

Iron, which is central to endocrine synthesis and regulation, has an ambivalent impact on reproductive health. Iron deficiency can lead to menstrual abnormalities [21,22,23,24,25] and affect gamete cell quality [26,27,28], while iron excess is associated with inflammatory conditions such as endometriosis [241,242,246], emphasizing the need for careful iron monitoring.

Copper is beneficial as a crucial antioxidant cofactor [304,305,306,307] for oocyte maintenance [307,308], but excess copper can trigger oxidative stress, highlighting the need to avoid copper-induced reproductive toxicity.

Collectively, these insights underscore the imperative for clinicians to recommend mineral supplementation with precision, tailored to individual health profiles, dietary patterns, and reproductive aspirations, to foster optimal reproductive well-being.

**Table 1:** Impact of Minerals of Essential Minerals on Fertility. Zn, Mg, Ca, I, Se, Fe, Cu, and Mn on key determinants of female fertility: Hormonal Regulation, Ovarian Function, Ovulation, Oxidative Stress, and Endometrial Implantation.

Mineral	Hormonal Regulation	Ovarian Function and Ovulation	Oxidative Stress	Endometrium and Implantation
Zn	Insulin metabolism [105] Steroid synthesis [39,40] Hormone Balance [37] Regulation of LH and FSH [38] Ovulation [219]	Oocyte maturation [208,209], development [228], and quality [226]	Antioxidant properties [264,269], protect embryos from ROS [43,44], and modulate inflammation in endometriosis [281,368]	Cellular proliferation and differentiation in the endometrium [56], neutralizes alterations [279] and deficiency correlates with endometrial cysts and polyps [47,48]
Mg	Cofactor for the production and function of estrogen [163,164], stabilizing glucose metabolism [174] and disbalances are associated with PCOS [165,166,168]	Mg deficiency leads to oxidative stress [327,329,330]. Magnesium can preserve the quality of the oocyte [251,327], modulate the ovulation process [338], and contribute to the healthy functioning of the endometrium [337].		Relax smooth muscle [54], influence retrograde menstruation [54,55], and reduce vascular endothelial growth factor, which may be beneficial in the treatment of gynecological conditions like endometriosis [57,58,59]
Ca	Calcium influences the release of GnRH, and therefore the menstrual cycle [29,30] Necessary for ovulation [396]	Oocyte activation and zygotic development [31,32], oocyte activation and fusion with sperm [33,34,35]	-	Calcium is deposited at the site of embryo implantation and regulates endometrial receptivity and embryo implantation [45,46]
I	Thyroid function and the synthesis of thyroid hormones, also reproductive hormones [62]. Deficiency [397], resulting in anovulation, reduced fertility, and menstrual cycle disturbances [65,66]	-	-	Improving endometrial receptivity [380,381,382] and supporting embryo implantation through endometrial changes [383,384,386]
Se	Thyroid function and thus important for hormone regulation [60,61] Thyroid disorders can lead to disrupted menstrual cycles and anovulation [63,64]	-	Supporting Glutathione Peroxidase (GPx) [263,290,293] Efficient ovulation and protection of the ovaries from damage [41,42]	-
Fe	Hormone synthesis and regulation of estrogen and progesterone [18,19,20] Deficiency can cause hormonal imbalances and affect menstrual cycles [21,22,23,24,25]	Maturation of oocytes [232,233,234,235,236] and cellular division during oocyte maturation [237] Deficiency leads to anemia, compromises the blood flow to the ovaries, and affects the quality of oocytes and the regularity of ovulation [26,27,28]	Deficiency and overdose lead to oxidative stress [262,314,315] and both reduce oocyte quality [24,102,236,323]	Deficiency impacts endometrial conditions and its receptiveness and decreases embryo implantation [49,50,51,52,53] Ferritin overdose is in correlation with endometriosis [241,242,246]
Cu	-	Cofactor for Superoxide Dismutase [266] and modulates antioxidant systems [304,305,306,307] Protects oocytes from oxidative stress [307,308] and supports endothelial function; crucial for optimal blood flow to the uterus and ovaries [309,310,311,312,313]		-
Mn	-	Cofactor for Superoxide Dismutase [341,342] and therefore supports oocyte quality and function [344,345] Overdose can contribute to oxidative stress and can damage oocyte and ovarian function [347,348,349]		-

Furthermore, the review provides actionable insights into appropriate mineral intake, offering guidelines for daily consumption based on different population groups—such as general women and pregnant women—and highlights the primary dietary sources for these minerals (Table 2). Such information is invaluable for clinicians who are tasked with advising women on how to optimize their reproductive health through nutrition.

In summary, the critical review and the data presented in Table 1 and Table 2 collectively contribute to a deeper understanding of mineral nutrition’s role in female fertility. They emphasize the importance of a balanced intake of essential minerals and provide a foundation for further research and clinical practice. 

## Figures and Tables

**Table 2:** Recommended daily mineral intake for women and primary dietary source. This table provides a comprehensive overview of several key minerals that are important for fertility (Zn, Fe, Se, Ca, Mg, I, Cu, and Mn). For each mineral, the table lists the recommended daily intake, the actual intake levels used in various studies, the main food sources, and the optimal serum levels. In this document, ‘N.A.’ signifies ‘Not Available’, which is used to indicate that specific information or data sought in the context of the study could not be obtained or was not accessible at the time of the research.

Mineral	Zn	Fe	Se	Ca	Mg	I	Cu	Mn
Recommended daily intake	8–11 mg [398]	18–27 mg [399]	55–60 µg [400]	1000–1200 mg [401]	320–350 mg [402]	70–130 µg [403]	1.3 mg [404]	3 mg [405,406]
Daily intake in studies	40 mg [407]–50 mg [72,408,409]	60 mg [102] 100 mg [410]–250 mg [410,411]	70 µg [412]	1000 mg [413,414]	250 mg [409,415]	60 μg [416]	1 mg [102]	3 mg
Main food source	Meat, dairy, nuts, whole grains [405]	Leafy Greens, Meat, Legumes [417]	Brazil nuts, fish, whole-wheat bread [418]	Dairy products, fortified foods, leafy greens [419]	Green leafy vegetables, nuts, seeds, whole grains	Brazil nuts, fish, whole-wheat bread [418]	Oyster, nuts, sunflower seeds, liver, whole grain, dark chocolate	Brown rice, hazelnuts, chickpeas, spinach, pumpkin seeds
Recommended serum levels	70–120 µg/dL [420]	15–150 ng/mL [421]	70–150 ng/mL [422]	8.6–10.2 mg/dL [419]	1.7–2.2 mg/dL [423]	100 µg/L (Urine)	70–150 µg/dL [424]	4–15 µg/L [405]
Optimal serum levels in studies	≥56 μg/dL [78]	N.A.	45 μg/L [425]–109 μg/L [426]	8.8–10.4 mg/dL [427] and <94 mg/L [428]	>1.4 mg/dL [429] 20.7 mg/L [428] 2.22–3.48 mg/dL [426]	79.9–138.5 μg/L [430] and >150 μg/L (urinary) [431]	88–177 μg/dL [405,426]	N.A.
